# Impact of low temperature on the efficacy of chlorous acid water and sodium hypochlorite solution for inactivating avian influenza virus on material surfaces

**DOI:** 10.1371/journal.pone.0360392

**Published:** 2026-10-26

**Authors:** Tomoko Morita, Abeer Mohamed Abdelfattah Elsayed, Rie Hirakawa, Basirat Mojisola Lawal-Ayinde, Tomoyuki Akita, Akima Yamamoto, Akifumi Higashiura, Toshihito Nomura, Isanori Horiuchi, Hisataka Goda, Junko Tanaka, Takemasa Sakaguchi

**Affiliations:** 1 Department of Virology, Graduate School of Biomedical and Health Sciences, Hiroshima University, Hiroshima, Japan; 2 Clinical Emerging Infectious Disease Laboratory, Hiroshima University Hospital, Hiroshima, Japan; 3 Department of Epidemiology, Infectious Disease Control, and Prevention, Graduate School of Biomedical and Health Sciences, Hiroshima University, Hiroshima, Japan; 4 Sankei Co., Ltd., Osaka, Japan; National Research and Innovation Agency, INDONESIA

## Abstract

Chlorous acid water is a type of chlorine-based disinfectant with chemical properties distinct from those of sodium hypochlorite solution, which is in common use. In this study, we evaluated the inactivation efficacy of chlorous acid water against avian influenza virus deposited on material surfaces, in comparison with sodium hypochlorite solution. First, the intrinsic antiviral activity of both disinfectants was examined under conditions free of amino acids and proteins. The results demonstrated that the 99% (IC_99_), 99.9% (IC_99.9_), and 99.99% inhibitory concentrations (IC_99.99_) were all approximately half those of sodium hypochlorite solution, indicating that chlorous acid water possesses slightly stronger inactivation activity. Next, the efficacy against protein-containing virus deposited on material surfaces (stainless steel, plastic, and rubber) was evaluated under conditions simulating a poultry house environment. At room temperature, both disinfectants exhibited equivalent inactivation capacity; however, a marked difference was observed under low-temperature conditions. Specifically, the efficacy of chlorous acid water was maintained or enhanced, whereas that of sodium hypochlorite solution declined, with the required concentration increasing approximately 2- to 3-fold. Taken together, these findings demonstrate that chlorous acid water exhibits antiviral activity comparable to or exceeding that of sodium hypochlorite solution, while retaining its efficacy even at low temperatures and in the presence of proteins. These properties suggest that chlorous acid water may be a particularly useful disinfectant in real-world settings such as poultry houses operating under low-temperature conditions.

## Introduction

Avian influenza viruses (AIV) are considered to be the principal reservoir of influenza viruses in nature, harboring all known subtypes. In particular, highly pathogenic strains, predominantly of the H5 and H7 subtypes, which possess a cluster of basic amino acids at the cleavage site of the hemagglutinin (HA) precursor protein, cause systemic infection in their avian hosts and exhibit marked virulence [1]. The impact on the poultry industry is severe; in Japan, when an outbreak occurs, measures to prevent viral spread, including the culling of all birds within the affected poultry house, are implemented, resulting in serious economic damage [2].

At present, sodium hypochlorite (NaClO) solution (SHS) is generally used for environmental disinfection in poultry houses and similar settings [3]. However, while sodium hypochlorite is a potent disinfectant, it has a significant drawback: upon contact with organic matter in the environment, such as proteins present in feces, blood, and feed, free available chlorine (FAC) is rapidly consumed, leading to a marked reduction in bactericidal and antiviral activity [4–8]. Furthermore, sodium hypochlorite reacts indiscriminately with a wide range of organic substances, and the risk of generating harmful mutagenic by-products, such as trihalomethanes, has also been noted [4,9].

As an alternative oxychloro disinfectant, chlorous acid water (CAW; principal active component: chlorous acid, HClO_2_) has attracted increasing attention in recent years [4]. Chlorous acid water produces minimal characteristic chlorine odor and is less prone to generating harmful substances such as trihalomethanes, conferring a high safety profile [4,9]. Its greatest distinguishing feature lies in its ability to react selectively with specific amino acids, including cysteine, histidine, tryptophan, and tyrosine, and with proteins, thereby destroying microorganisms and viruses through oxidative denaturation of proteins [5,10]. Because of this limited reactivity with organic matter, FAC is more readily maintained even under challenging protein-rich conditions, and CAW has been reported to exert stable, sustained inactivation activity against a broad range of pathogens, including human norovirus, feline calicivirus, SARS-CoV-2, and *Clostridium difficile* spores, surpassing the efficacy of sodium hypochlorite [4–9,11].

In the present study, we compared and evaluated the disinfection efficacy of SHS and CAW against AIV under conditions simulating actual poultry house disinfection: namely, virus deposited on material surfaces and low-temperature conditions representative of the winter season.

## Materials and methods

### Ethics statement

No ethical approval was necessary, as the present study had no human participants, and the authors did not perform any procedure on animals.

### Reagents

Chlorous acid water (Klorus Acid・N barrier) was provided by Sankei Co., Ltd. (Osaka, Japan). Sodium hypochlorite solution (PURELOX-S) was purchased from Oyalux Co., Ltd. (Tokyo, Japan). Fetal bovine serum (FBS) was purchased from BIOSERA (Boussens, France).

The free available chlorine (FAC) concentrations of the chlorous acid water and the sodium hypochlorite solution were measured immediately before each experiment by the DPD method using an RC-V2-CAW meter (Kasahara Chemical Instruments Corp., Saitama, Japan) [6]. The stock solutions were diluted with sterile distilled water, the FAC values were measured, and the experiments were performed based on these values. The stock solutions were stored in glass containers, and polystyrene tubes were used for dilution to prevent inactivation of the chlorine-based disinfectants.

### Test plates for surface tests

Plates of stainless steel (SUS316L 2B), plastic (polypropylene, KOBE POLYSHEET PP), and rubber (nitrile rubber, NBR-70), each with dimensions of 50 × 50 × 1 mm, were purchased from Standard Testpiece Co., Ltd. (Kusatsu, Japan).

### Cells and virus

MDCK(+) cells derived from canine kidney epithelial cells [12] were cultured in Dulbecco’s modified Eagle’s medium (DMEM; FUJIFILM Wako Pure Chemical Corporation, Osaka, Japan) supplemented with 5% FBS, 100 units/mL penicillin G (Meiji Seika Pharma Co., Ltd., Tokyo, Japan), and 100 μg/mL streptomycin (Meiji Seika Pharma) at 37 °C under 5% CO_2_.

Avian influenza virus (AIV) A/whistling swan/Shimane/499/83 (H5N3) was provided by Dr. Otsuki (Tottori University, Japan). To obtain purified virus, the virus was inoculated into embryonated chicken eggs and propagated [13]. After removal of cellular components from the infected allantoic fluid by low-speed centrifugation, the fluid was layered over a 7 mL cushion of 20% (w/w) sucrose in phosphate-buffered saline (PBS) and ultracentrifuged at 22,000 rpm (approximately 83,000 × g) for 2 h at 4 °C using a Beckman SW-32Ti rotor. The resulting virus pellet was suspended in physiological saline, aliquoted, and stored at −80 °C until use.

### Antiviral tests

After measuring the FAC concentrations of the chlorous acid water and the sodium hypochlorite solution, the reagents were diluted in polystyrene tubes to prepare the prescribed concentrations [7].

The suspension test, in which the disinfectant solution and the virus suspension are mixed, was performed according to a previously reported method based on a partial modification of the ISO 14476 method [14]. Virus suspension (10 μL) was added to each test reagent (90 μL) and allowed to react for the prescribed time at room temperature (approximately 25 °C). The reaction was then stopped by 10-fold dilution with DMEM containing 10% FBS and 10 μM sodium thiosulfate. This mixture was serially diluted 10-fold with DMEM containing 10% FBS, and each dilution was inoculated onto MDCK(+) cells in a 96-well plate (4–8 wells per dilution). After inoculation, the cells were cultured in a CO_2_ incubator for approximately 5 days until the cytopathic effect (CPE) was fully developed. The CPE was observed under a microscope, and the infectious titer, expressed as the 50% tissue culture infectious dose (TCID₅₀/mL), was calculated by the Behrens–Kärber method [15].

The disinfection efficacy on material surfaces was evaluated using a partial modification of the ISO 21702 method (Fig 1). After each plate surface was disinfected by UV irradiation, 50 µL of virus suspension containing 5% FBS was applied as several separate droplets distributed within the central 4 × 4 cm area of the 5 × 5 cm plate and dried under a sterile airflow in a safety cabinet. After drying, 200 µL of chlorous acid water (CAW) or sodium hypochlorite solution (SHS) was added and covered with a PE film (4 × 4 cm) to spread the liquid uniformly as a thin layer covering the entire inoculated area, so that the whole of the dried virus deposit was in contact with the disinfectant throughout the contact time. After incubation at room temperature for 10 min, the PE film was removed, and 200 µL of virus recovery solution (DMEM containing 10% FBS and 10 µM sodium thiosulfate) was added to recover the virus by pipetting. The infectious titer in the recovered solution was then measured by the TCID₅₀ method.

**Fig 1:**
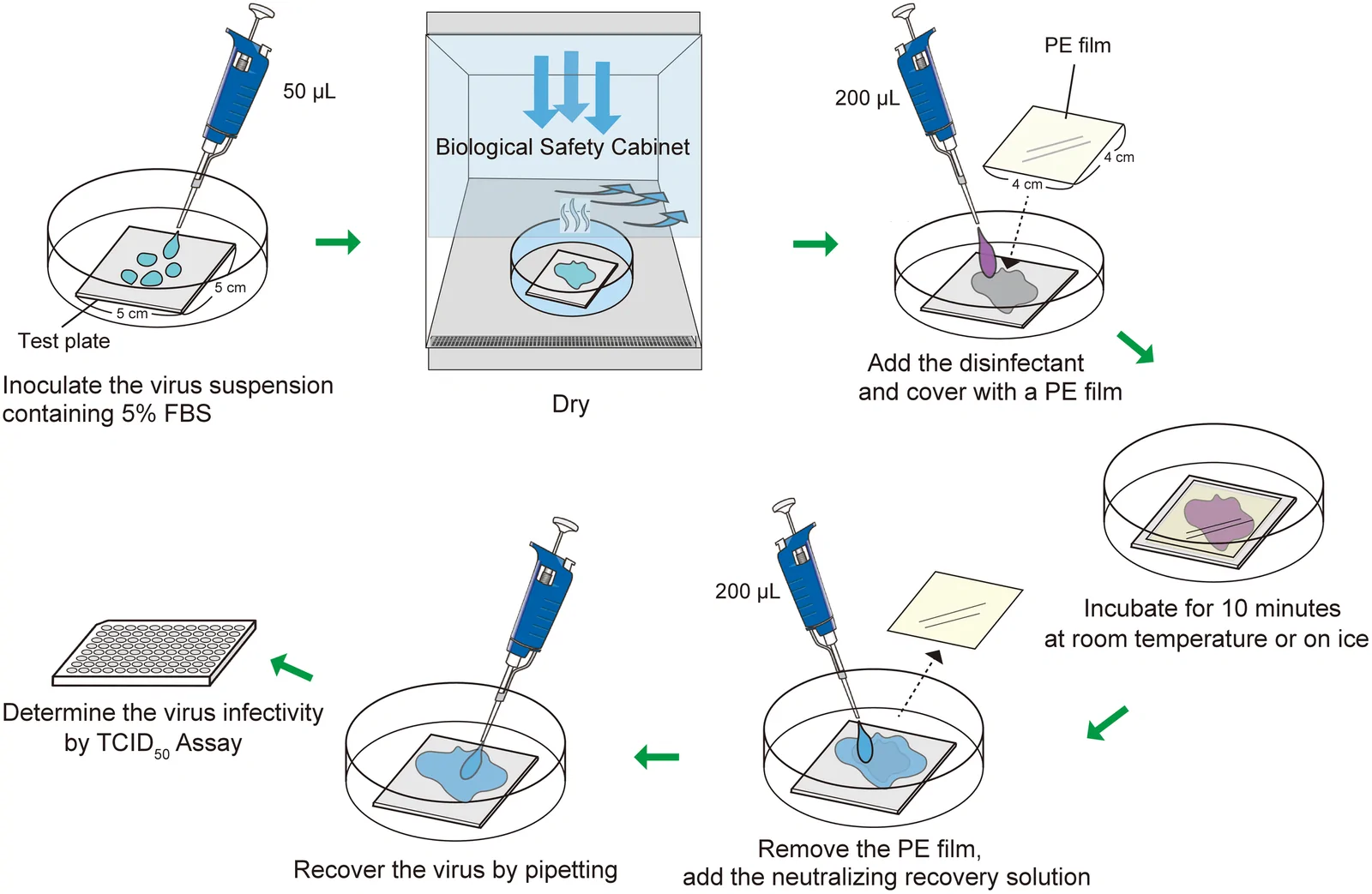
Experimental procedure for the surface test. Performed according to the method described in the antiviral test section of the Materials and Methods.

### Statistical analysis

Infectivity was plotted against disinfectant concentration, and approximation curves were generated by four-parameter logistic regression using JMP (version 19.0.4; SAS Institute, Cary, NC, USA). Based on these curves, the 99% inhibitory concentration (IC₉₉), 99.9% inhibitory concentration (IC₉₉.₉), and 99.99% inhibitory concentration (IC₉₉.₉₉) were calculated for each condition. For graph plotting, a smoothing spline method with the default smoothing parameter was applied.

## Results

### Inactivation efficacy and rapidity of chlorous acid water in the absence of amino acids

Previous studies have demonstrated that the inactivation activity of chlorous acid water (CAW) and sodium hypochlorite solution (SHS) against SARS-CoV-2 is influenced by the presence of contaminating proteins or amino acids [8].

In the present study, to assess the intrinsic inactivation efficacy of both chlorine-based disinfectants against AIV, purified virus preparations — from which medium-derived amino acids and proteins such as fetal bovine serum (FBS) had been removed by ultracentrifugation — were used for evaluation.

When the virus was reacted with each disinfectant at room temperature for one minute, CAW was found to inactivate the purified virus at lower concentrations than SHS (Fig 2A, panels A and B in S1 Fig). Calculation of the 99% (IC_99_), 99.9% (IC_99.9_), and 99.99% inhibitory concentrations (IC_99.99_) revealed that the IC values for SHS were more than twice those of CAW in all cases (Table 1). These results indicate that CAW possesses inactivation activity against purified virus that is equivalent to or greater than that of SHS.

**Table 1:** Inactivation effects of chlorous acid water and sodium hypochlorite solution on purified avian influenza virus particles.

	CAW	Infectivity	SHS	Infectivity
	(mg/L)	(TCID_50_/mL)	(mg/L)	(TCID_50_/mL)
No reagent	0	1.1 × 10^7^	0	4.7 × 10^7^
99% Inhibition (IC_99_)	1.3	1.1 × 10^5^	2.8	4.7 × 10^5^
99.9% Inhibition (IC_99.9_)	1.5	1.1 × 10^4^	3.6	4.7 × 10^4^
99.99% Inhibition (IC_99.99_)	1.6	1.1 × 10^3^	4.5	4.7 × 10^3^

**Fig 2:**
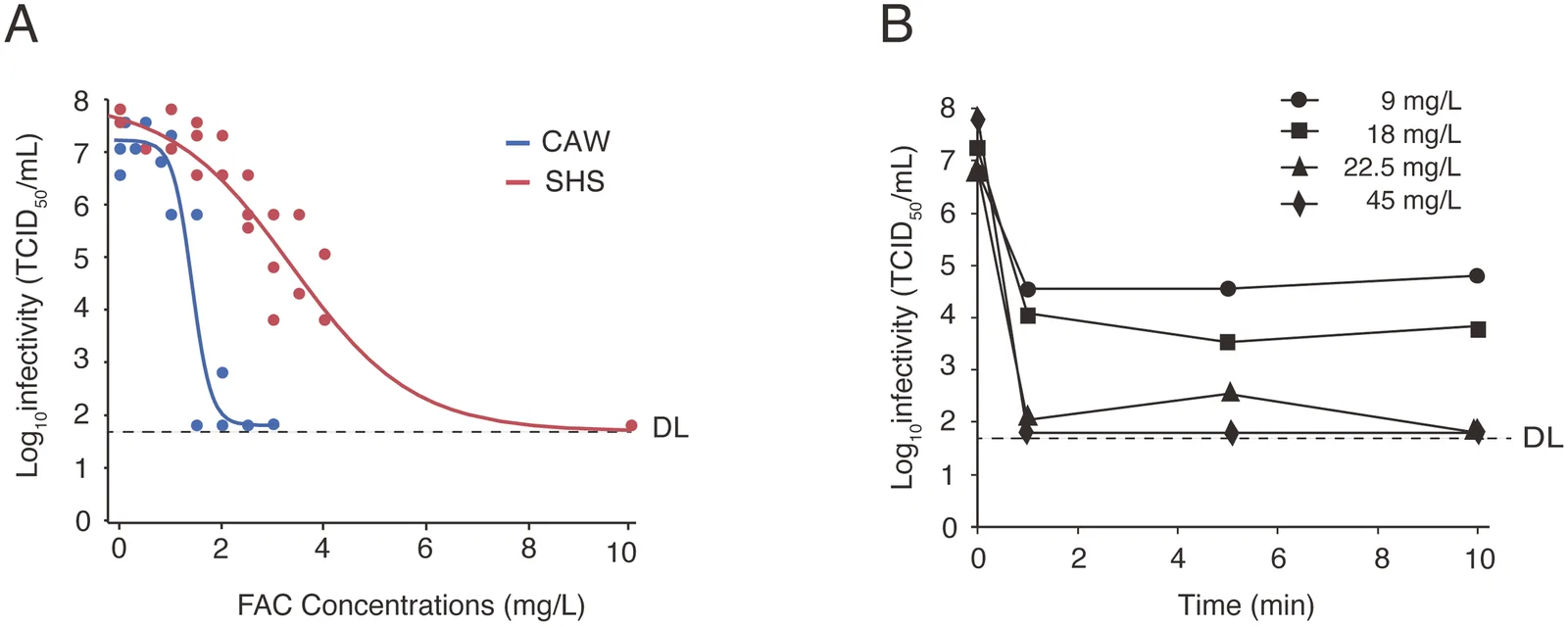
Effect of chlorine-based disinfectants on purified influenza virus particles. (A) Avian influenza virus was propagated in embryonated chicken eggs and then highly purified by ultracentrifugation. The purified virus was treated for 1 min with chlorous acid water (CAW; blue) or sodium hypochlorite solution (SHS; red) at various concentrations. After the reaction was stopped, the viral infectious titer was measured. Drug concentration and viral infectious titer were plotted on a graph, and an approximation curve was fitted by four-parameter logistic regression using JMP. (B) The purified virus was treated with chlorous acid water (CAW) at various concentrations for 1, 5, and 10 min, and the residual viral infectious titer was measured after the reaction was stopped. The concentrations used are indicated in the graph. DL: detection limit.

Purified avian influenza virus particles (A/whistling swan/Shimane/499/83 (H5N3)) were incubated with either chlorous acid water (CAW) or sodium hypochlorite solution (SHS) at room temperature for 1 minute, and the residual infectivity was measured. Based on the obtained data (S1 Fig), the 99% inhibitory concentration (IC_99_), 99.9% inhibitory concentration (IC_99.9_), and 99.99% inhibitory concentration (IC_99.99_) were calculated.

Furthermore, time-course analysis of virus inactivation by CAW revealed that, at all concentrations tested (9–45 mg/L), viral infectivity decreased to the minimum value observed at each disinfectant concentration within one minute of the start of the reaction. At 22.5 mg/L and 45 mg/L, infectivity declined below the limit of detection, confirming nearly complete inactivation (Fig 2B).

### Efficacy of chlorine-based disinfectants against virus deposited on material surfaces at room temperature

Next, to evaluate the practical utility of these disinfectants under conditions simulating real-world environments such as poultry houses, the disinfection efficacy was assessed against virus suspended in 5% FBS — used as an organic load — that had been deposited and dried on material surfaces (stainless steel, plastic, and rubber). The inclusion of FBS was intended to prevent the tight adsorption of virus to material surfaces and to ensure adequate recovery rates.

First, a suspension test was performed in which the virus suspension containing 5% FBS was reacted directly with each disinfectant at room temperature for 10 minutes without prior deposition onto any material surface. Both CAW and SHS effectively inactivated the virus under these conditions, and the IC_99_, IC_99.9_, and IC_99.99_ values for the two disinfectants were nearly equivalent (Fig 3, Table 2: “No Material”, panels C and D in S1 Fig).

**Table 2:** Inactivation effects of chlorous acid water and sodium hypochlorite solution on avian influenza virus on material surfaces.

Material		CAW (mg/L)			SHS (mg/L)		
	Inhibition	RT	on ice	Ratio	RT	on ice	Ratio
				([on ice]/ RT)			([on ice]/ RT)
Stainless Steel	IC_99_	58.0	49.5	0.9	128.6	334.1	2.6
	IC_99.9_	86.8	50.4	0.6	158.2	413.0	2.6
	IC_99.99_	122.4	51.5	0.4	186.8	535.8	2.9
Plastic	IC_99_	74.6	32.1	0.4	143.4	405.1	2.8
	IC_99.9_	99.9	46.3	0.5	183.1	464.3	2.5
	IC_99.99_	129.8	65.6	0.5	213.9	516.9	2.4
Rubber	IC_99_	102.3	31.0	0.3	157.1	440.0	2.8
	IC_99.9_	136.8	43.2	0.3	233.7	497.4	2.1
	IC_99.99_	170.4	59.1	0.3	313.9	538.1	1.7
No Material	IC_99_	13.7	17.6	1.3	14.2	23.9	1.7
	IC_99.9_	21.8	21.2	1.0	22.9	40.2	1.8
	IC_99.99_	31.9	25.1	0.8	34.4	64.1	1.9

An avian influenza virus solution containing 5% FBS was dropped onto material surfaces (stainless steel, plastic, or rubber) and dried. Chlorous acid water (CAW) or sodium hypochlorite solution (SHS) was added, covered with PE film, and incubated for 10 minutes either at room temperature (RT: approx. 25°C) or on ice (0°C). The PE film was then removed, a virus recovery solution was added, and the virus was recovered by pipetting to measure its infectivity. Based on the obtained data, the 99% inhibitory concentration (IC_99_), 99.9% inhibitory concentration (IC_99.9_), and 99.99% inhibitory concentration (IC_99.99_) were calculated. Additionally, the ratio of IC values between [on ice] and RT (Ratio), as well as the results of the test conducted by directly reacting the virus solution with the disinfectant without adherence to any material (No Material), are also shown.

**Fig 3:**
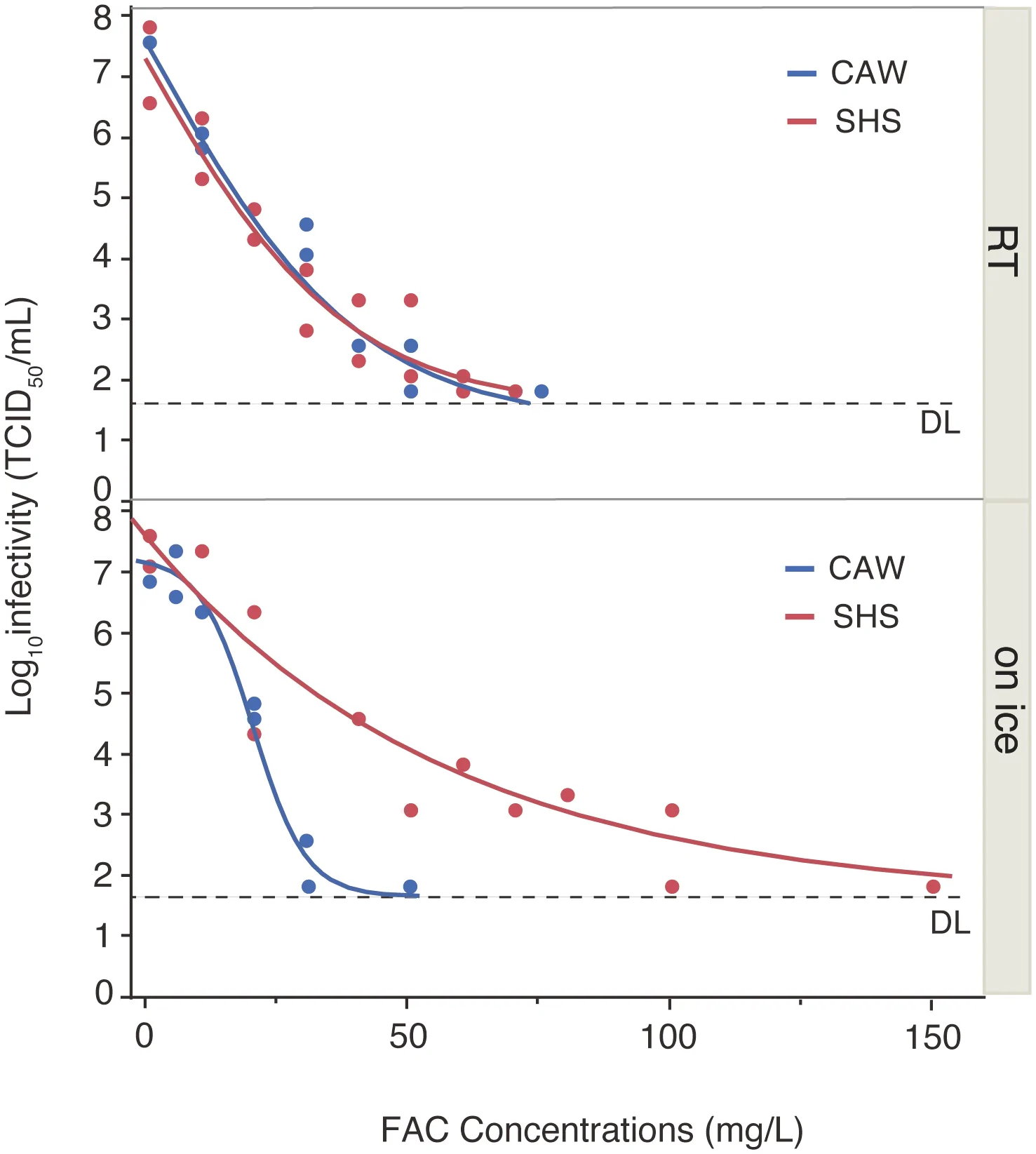
Effect of chlorine-based disinfectants on the viral suspension used in the surface test. Ten µL of the viral suspension used in the surface test (containing 5% FBS) was mixed with 90 µL of chlorous acid water (CAW; blue) or sodium hypochlorite solution (SHS; red) at various concentrations, and the mixture was incubated for 10 min at room temperature (RT) or on ice. The residual infectious titer was measured. Drug concentration and viral infectious titer were plotted on a graph, and an approximation curve was fitted by four-parameter logistic regression using JMP. DL: detection limit.

Next, each disinfectant was applied to virus deposited on material surfaces and allowed to react at room temperature for 10 minutes (Fig 4). Compared with the suspension test (no material), the IC values for CAW increased 4- to 6-fold and those for SHS increased 7- to 10-fold in the surface deposition tests (Table 2, panels A–F in S2 Fig), indicating that deposition of virus onto material surfaces reduced disinfectant efficacy by up to approximately 10-fold.

**Fig 4:**
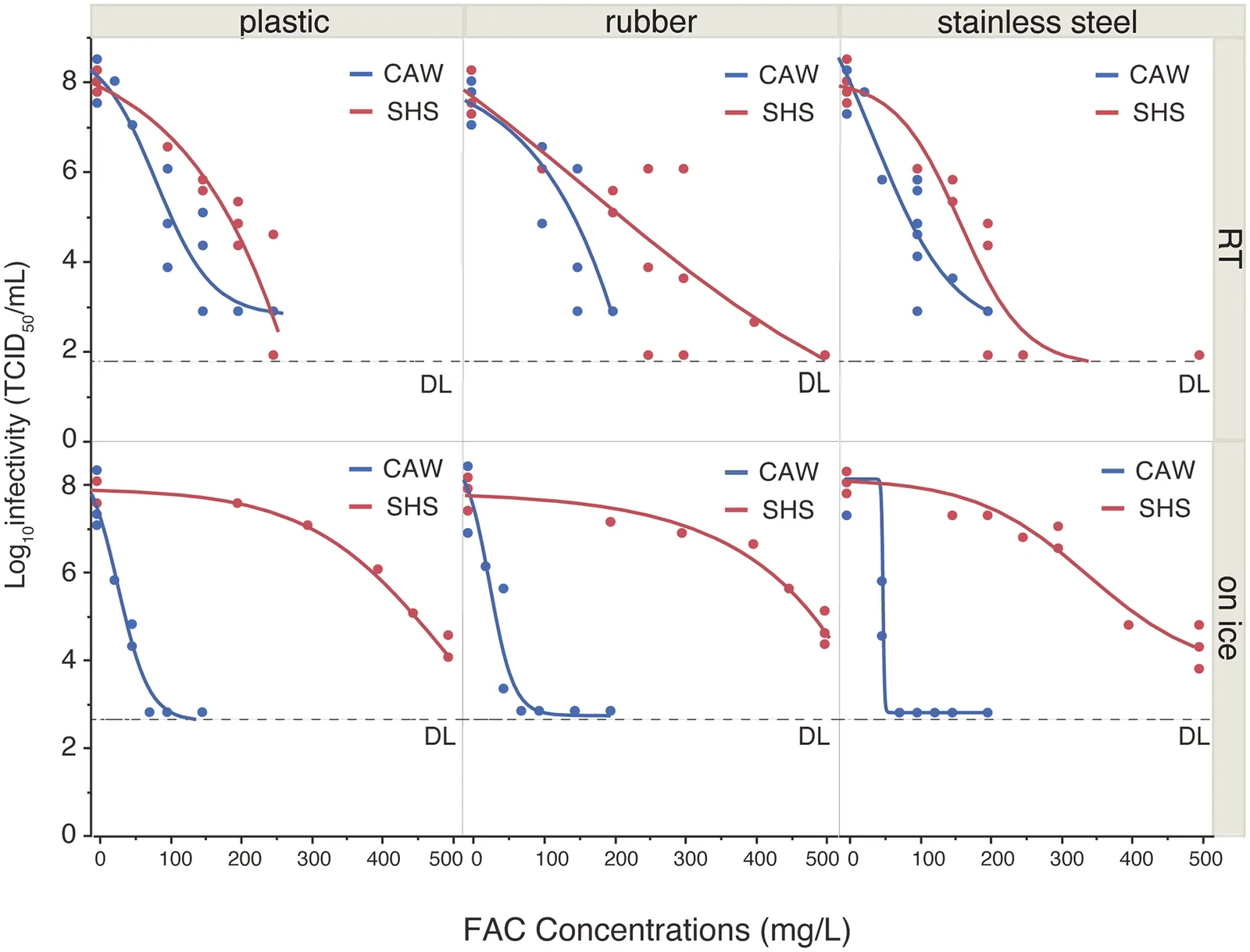
Surface test. Viral suspension containing 5% FBS was applied to plates of plastic (polypropylene), rubber (nitrile rubber), or stainless steel and allowed to dry. Then, chlorous acid water (CAW; blue) or sodium hypochlorite solution (SHS; red) at various concentrations was added, and the plates were incubated for 10 min at room temperature (RT) or on ice. A recovery solution containing reaction stoppers (sodium thiosulfate, FBS, DMEM) was added to suspend the virus, and its infectious titer was measured. Drug concentration and viral infectious titer were plotted on a graph, and an approximation curve was fitted by four-parameter logistic regression using JMP. DL: detection limit.

Comparison among materials revealed a tendency for IC values to increase — and inactivation efficiency to decrease — in the order of stainless steel, plastic, and rubber, for both CAW and SHS treatments. However, the differences were within approximately 2-fold at most, and no marked dependence on material type was observed (Fig 4, Table 2).

Across all surface deposition tests conducted at room temperature, the IC values for CAW were consistently lower than those for SHS, suggesting a slightly superior inactivation efficacy (Table 2). Taken together, these results confirm that CAW exhibits efficacy comparable to or exceeding that of SHS even under conditions involving virus deposited on organic matter-laden material surfaces.

### Inactivation efficacy of chlorine-based disinfectants under low-temperature conditions

To simulate winter poultry house conditions, disinfection efficacy was evaluated under low-temperature conditions in which reactions were carried out on ice for 10 minutes. In the suspension test, in which the virus suspension containing 5% FBS was reacted directly with each disinfectant on ice for 10 minutes, IC values increased 2- to 3-fold relative to room temperature conditions for both disinfectants, indicating a reduction in inactivation capacity (Fig 3, Table 2, panels E and F in S1 Fig).

Subsequently, each disinfectant was applied to virus-deposited plates and allowed to react under low-temperature conditions. For SHS, IC values under low-temperature conditions increased to approximately 2- to 3-fold those at room temperature, reflecting a reduction in inactivation capacity (Fig 4, Table 2: Ratio [on ice]/RT, panels G–L in S2 Fig). In contrast, CAW showed no reduction in efficacy at low temperature; on the contrary, IC values decreased to 0.3- to 0.9-fold those at room temperature, indicating a tendency toward enhanced inactivation activity (Fig 4, Table 2: Ratio [on ice]/RT).

These results demonstrate that whereas the inactivation efficacy of SHS is diminished under low-temperature conditions in both the suspension and surface deposition tests, CAW does not exhibit a reduction in efficacy at low temperature.

## Discussion

This study evaluated the inactivation efficacy of chlorous acid water (CAW) against AIV under conditions simulating real-world environments such as poultry houses and compared and validated it against the widely used sodium hypochlorite solution (SHS). The results demonstrated that CAW not only exhibits antiviral activity equal to or greater than that of SHS but also maintains its superiority under conditions approximating real-world environments, including organic matter loading, material surfaces, and low-temperature settings.

In validation using purified virus free of organic matter, CAW achieved equivalent inactivation capacity at approximately half the inhibitory concentration (IC value) of SHS. This indicates that CAW possesses more potent antiviral activity as an intrinsic disinfectant. Furthermore, time-course analysis confirmed rapid inactivation, with infectivity titers falling below the detection limit within one minute of the reaction onset. CAW has been reported to exhibit strong, rapid inactivation effects against other viruses as well, including feline calicivirus (a norovirus surrogate) and SARS-CoV-2 [4,5,16], and the present study demonstrated that it possesses similar fast-acting efficacy against AIV.

In actual environments such as poultry houses, viruses adhere to material surfaces together with organic matter (proteins) such as feces, blood, and feed. In this study, when virus suspensions containing 5% FBS were adhered to and dried on stainless steel, plastic, and rubber surfaces, the IC values of both disinfectants increased by up to approximately tenfold compared with the suspension test, indicating attenuated efficacy. This is thought to result from the physical impediment of drug penetration caused by viral adhesion to the material surfaces. In the comparison among materials, IC values increased in the order of stainless steel, plastic, and rubber, showing a tendency toward decreased inactivation efficiency; however, the difference remained within twofold at most, indicating limited dependence on the material. Throughout the surface-adhesion tests conducted at room temperature, CAW exhibited lower IC values than SHS, maintaining high inactivation efficacy equal to or greater than that of SHS even under conditions approximating real-world environments, namely material surfaces with organic matter loading.

The most important finding of this study is the clear difference in the behavior of the two disinfectants under low-temperature conditions (on ice) simulating a winter environment. When the ratio of low-temperature to room-temperature IC values (low-temperature/room-temperature ratio) was calculated, SHS showed an increase of approximately two- to threefold, indicating reduced inactivation capacity. Even in the suspension test, where no material surface was present, the efficacy of SHS decreased by approximately twofold at low temperature, which is thought to be attributable to the intrinsic physicochemical properties of SHS. Since chemical reaction rates generally decrease with declining temperature, this result is consistent with thermodynamic theory. In contrast, for CAW the IC value ratio decreased to 0.3–0.9-fold, yielding the opposite result in which inactivation efficacy was rather enhanced. This mechanism suggests that CAW may possess distinctive physicochemical properties, retaining high penetrability and chemical reactivity toward virus particles on the solid phase even at low temperatures.

The efficacy of CAW depends on the chemical speciation of the solution. Free chlorous acid (HClO_2_) is thermodynamically unstable and disproportionates to chlorine dioxide, chlorate and hypochlorous acid; the reaction is second order in HClO_2_ and is strongly accelerated by acidity, while chloride exerts both catalytic and inhibitory effects, that is, it may either accelerate or suppress the reaction depending on its concentration and on the pH [17]. The active species present in an unbuffered chlorous acid solution can therefore change appreciably over a relatively short period. The CAW used in the present study, however, is not free chlorous acid in water but a formulation in which chlorous acid is maintained in equilibrium with its conjugate base, chlorite, under weakly acidic conditions; storage conditions that stabilize chlorous acid in aqueous solution have been described previously [18]. In this study the FAC concentration of every solution was determined by the DPD method immediately before each experiment, and the working dilutions were prepared immediately before use, so that the IC values reported here correspond to the FAC actually present at the moment of contact with the virus. The slower disproportionation expected at reduced temperature offers a plausible chemical explanation for the retention, and apparent enhancement, of CAW activity on ice, in contrast to the loss of activity of SHS under the same conditions.

From a practical standpoint, CAW is supplied as a stabilized stock solution that is diluted with water at the site of use. The supplier recommends that the stock solution be stored unopened for no longer than approximately one year. Once opened, it should be kept in a light-resistant, airtight container in a cool, dark place and used within a few months. Because the FAC of any chlorine-based solution declines with time and on contact with organic matter, we recommend (i) that the working solution be prepared in the volume required for a single disinfection session and used within 7 days of preparation, (ii) that the stock solution be kept in a closed, light-protected container with the headspace kept to a minimum, and (iii) that the FAC of the working solution be confirmed at the point of use by a simple field method such as the DPD method employed here or the tetramethylbenzidine method [6] developed for chlorite-based sanitizers in organic-matter-rich environments. Two practical advantages of CAW follow from the present results. First, because the FAC of CAW is consumed more slowly by organic matter than that of SHS [4–8], a working solution of CAW retains its activity better in the heavily soiled environment of a poultry house, which partly offsets its lower intrinsic chemical stability. Second, because the efficacy of CAW is not reduced at low temperature, the working concentration does not need to be raised in winter, whereas the concentration of SHS would have to be increased approximately two- to threefold to achieve the same degree of inactivation under the same conditions. Against these advantages must be set the lower intrinsic stability of chlorous acid relative to hypochlorite, which imposes practical constraints on storage and distribution and makes verification of FAC at the point of use more important than it is for SHS.

Two limitations of the present study should be noted. First, the efficacy measured in a surface test depends not only on inactivation of the virus but also on the efficiency with which residual infectious virus is recovered from the material, so a contribution of the disinfectants themselves to the recovery step cannot be strictly excluded. Three observations nevertheless argue that the difference between CAW and SHS is not an artifact of recovery. (i) An identical recovery procedure, using a recovery solution containing a neutralizer, was applied to both disinfectants and to the untreated controls on every material. (ii) The greater intrinsic potency of CAW was already apparent in the suspension test with purified virus, in which virus and disinfectant were simply mixed and neither a material surface nor a recovery step was involved (Table 1; Fig 2A; panels A and B in S1 Fig). (iii) The temperature dependence that constitutes the principal finding of this study, namely retention of CAW activity together with loss of SHS activity on ice, was likewise reproduced in the suspension test in the absence of any material surface (Fig 3; Table 2, “No Material”; panels E and F in S1 Fig). Direct quantification of recovered viral genome copies by qPCR would allow recovery efficiency to be assessed independently of infectivity and would be a useful addition to future surface-disinfection studies; because qPCR does not distinguish infectious from inactivated particles, however, it would complement rather than replace the infectivity measurements used here. Second, 5% FBS was used as the organic soil because it provides a standardized and reproducible protein load comparable with that of our earlier studies, and because it was necessary in order to recover virus efficiently from the material surfaces. Serum proteins promote elution far more strongly than the dried fecal, blood and feed residues encountered in a poultry house, and evaluation against such field-relevant soils will therefore be required before the concentrations reported here can be applied directly to practical disinfection protocols.

Outbreaks of avian influenza are concentrated predominantly in the winter season, and actual poultry houses represent environments characterized by both low temperatures and the abundant presence of organic matter. Taken together, these findings strongly suggest that CAW has the potential to serve as an exceptionally useful and reliable disinfectant for the prevention of AIV transmission in the poultry industry, surpassing the performance of conventional SHS.

## Supporting information

S1 Fig Concentration–response data and four-parameter logistic regression curves for the suspension tests. Dose–response data used to calculate the IC_99_, IC_99.9_, and IC_99.99_ values shown in Table 1 and in Table 2 (“No Material”). (A, B) Purified avian influenza virus treated with chlorous acid water (A) or sodium hypochlorite solution (B) for 1 min at room temperature, in the absence of added protein. (C, D) Virus suspension containing 5% FBS treated with chlorous acid water (C) or sodium hypochlorite solution (D) for 10 min at room temperature. (E, F) Virus suspension containing 5% FBS treated with chlorous acid water (E) or sodium hypochlorite solution (F) for 10 min on ice. Each panel shows the raw infectivity data, the fitted curve, the four parameters (a–d), and the coefficient of determination (R^2^). Curves were fitted using JMP.
(PDF)

S2 Fig Concentration–response data and four-parameter logistic regression curves for the surface tests. Dose–response data used to calculate the IC values for virus deposited on material surfaces (Table 2). (A–F) Reactions at room temperature; (G–L) reactions on ice. Each pair shows chlorous acid water (A, C, E, G, I, K) or sodium hypochlorite solution (B, D, F, H, J, L) on stainless steel (A, B, G, H), plastic (C, D, I, J), and rubber (E, F, K, L). Each panel shows the raw infectivity data, the fitted curve, the four parameters (a–d), and the coefficient of determination (R^2^). Curves were fitted using JMP.
(PDF)
