# Supplementary material for: Impact of low temperature on the efficacy of chlorous acid water and sodium hypochlorite solution for inactivating avian influenza virus on material surfaces
Source: PLoS One. 2026 Oct 26;21(10):e0360392. doi: 10.1371/journal.pone.0360392 (PMC13704805; doi:10.1371/journal.pone.0360392)
Supplement: Supplementary file 1 [file pone.0360392.s001.pdf]

A Chlorous acid no protein RT

Conc. (mg/L)	Infectivity (TCID50/mL)
0	4.E+07
0	4.E+06
0	1.E+07
0.1	4.E+07
0.3	1.E+07
0.5	4.E+07
0.8	6.E+06
1	2.E+07
1	6.E+05
1	2.E+07
1.5	6.E+05
1.5	6.E+01
2	6.E+02
2	6.E+01
2.5	6.E+01
2.5	6.E+01
3	6.E+01
3	6.E+01
3	6.E+01

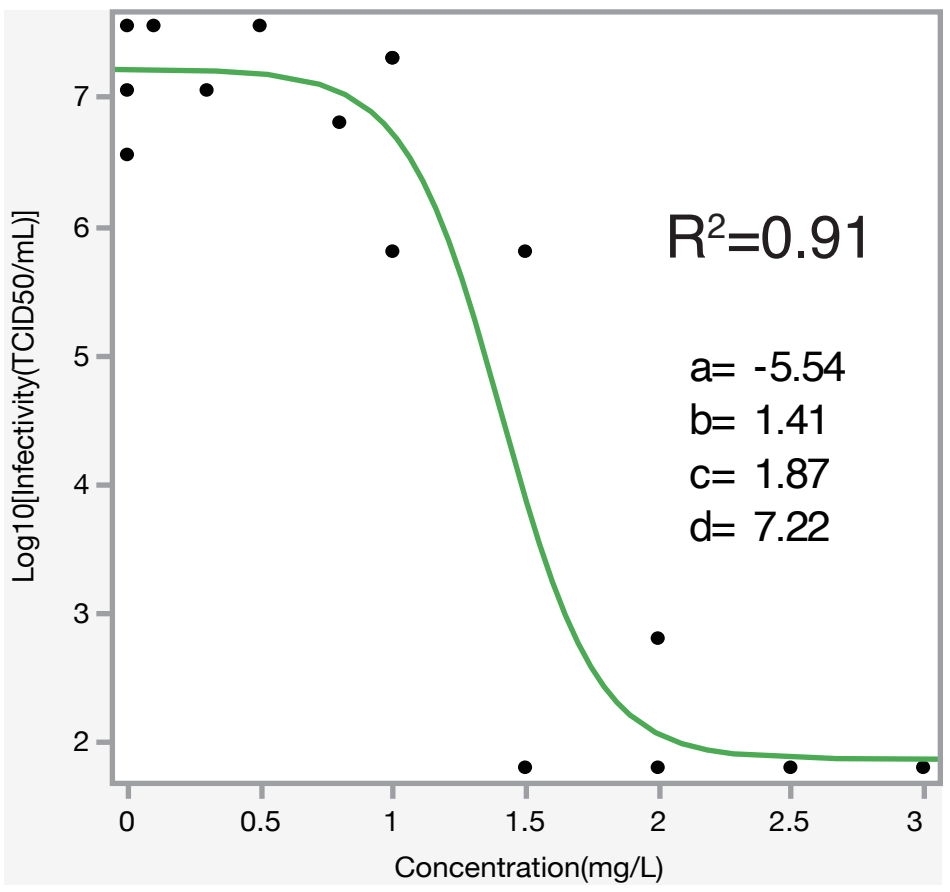

B Sodium hypochlorite no protein RT

Conc. (mg/L)	Infectivity (TCID50/mL)
0	6.E+07
0	6.E+07
0	4.E+07
0	4.E+07
0.5	1.E+07
1	6.E+07
1	1.E+07
1	6.E+05
1.5	4.E+06
1.5	2.E+07
1.5	4.E+07
2	4.E+06
2	2.E+07
2	2.E+07
2.5	4.E+05
2.5	6.E+05
2.5	4.E+06
3	6.E+03
3	6.E+05
3	6.E+04
3.5	6.E+05
3.5	2.E+04
4	1.E+05
4	6.E+03
10	6.E+01

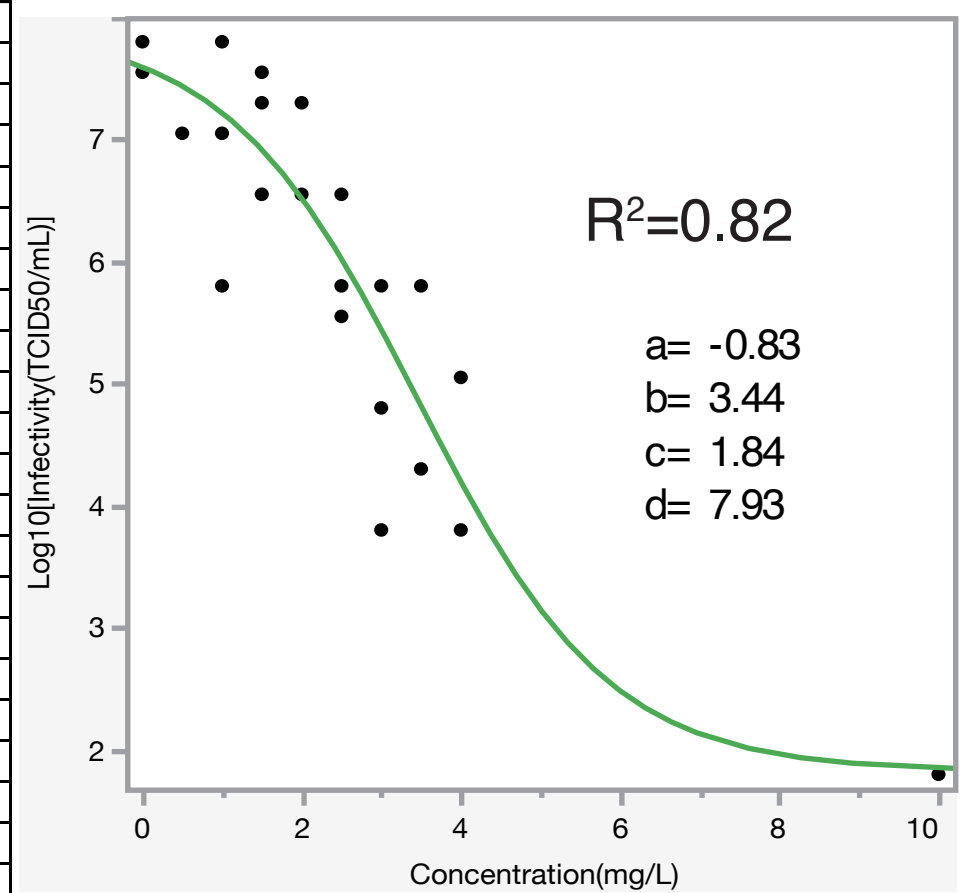

C Chlorous acid 5%FBS RT

Conc. (mg/L)	Infectivity (TCID50/mL)
0	4.E+07
0	4.E+07
10	1.E+06
10	6.E+05
20	6.E+04
20	2.E+04
30	4.E+04
30	1.E+04
40	4.E+02
50	4.E+02
50	6.E+01
60	6.E+01
75	6.E+01
75	6.E+01

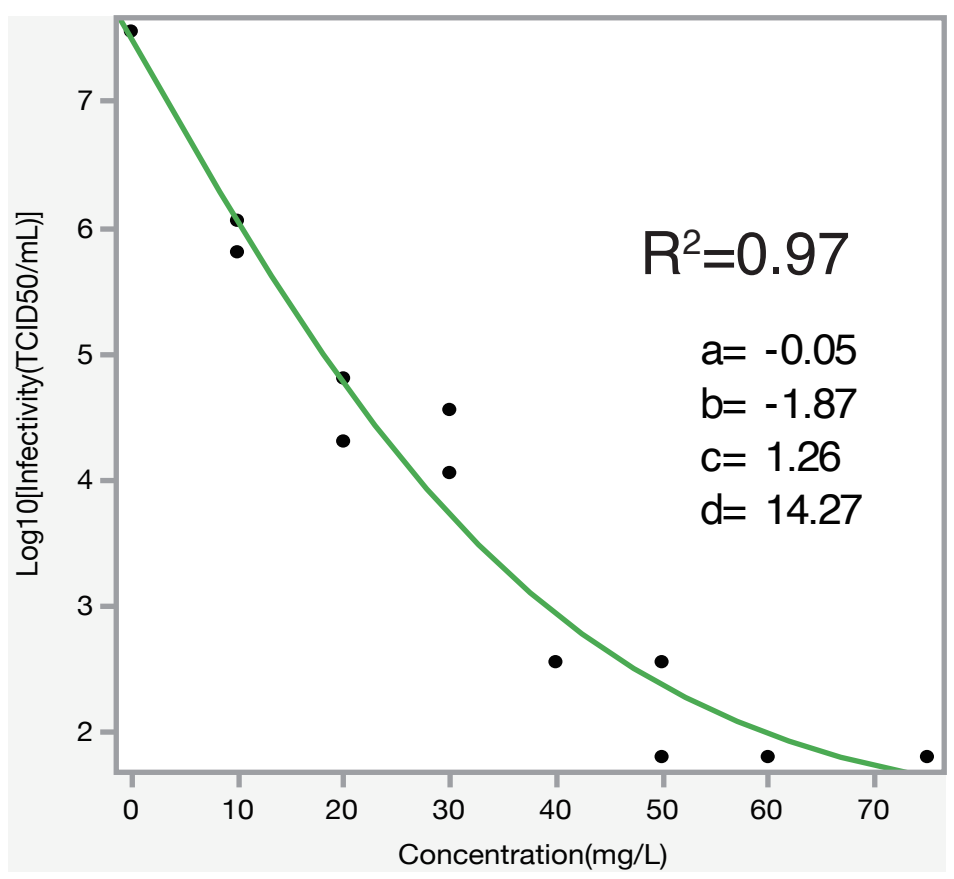

D Sodium hypochlorite 5%FBS RT

Conc. (mg/L)	Infectivity (TCID50/mL)
0	6.E+07
0	4.E+06
10	2.E+06
10	2.E+05
20	6.E+04
20	2.E+04
30	6.E+03
30	6.E+02
40	2.E+03
40	2.E+02
50	2.E+03
50	1.E+02
60	1.E+02
60	6.E+01
70	6.E+01
70	6.E+01

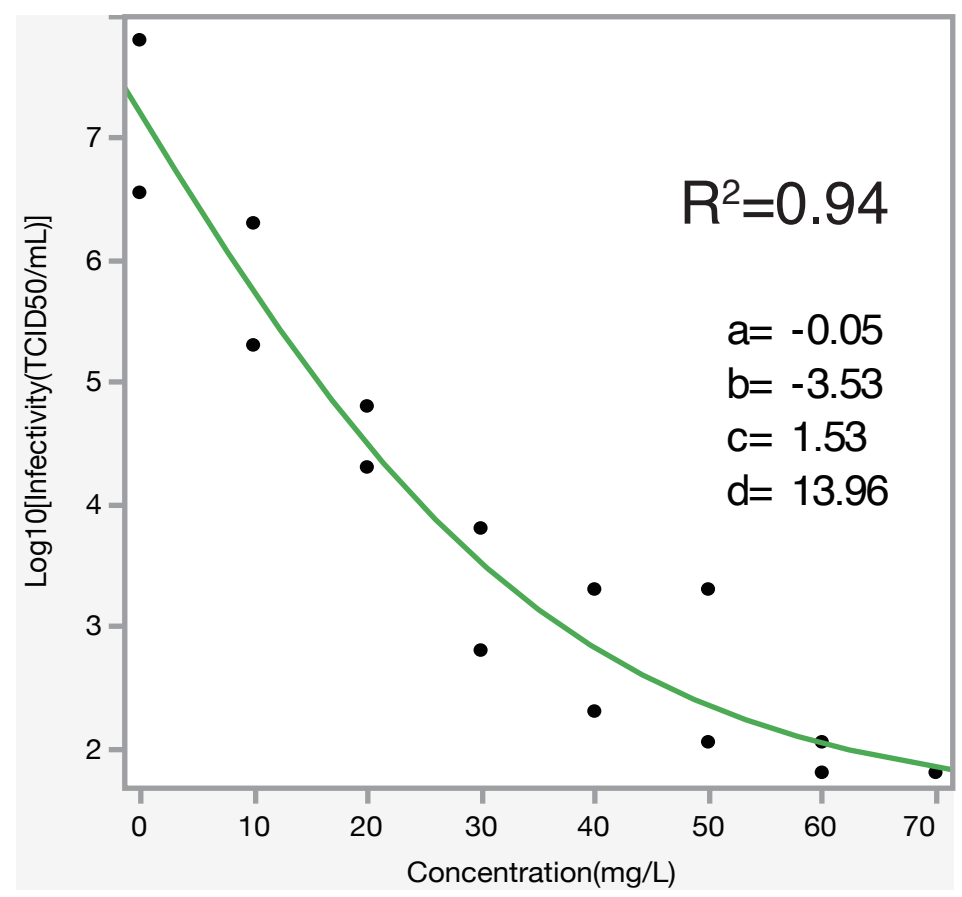

E Chlorous acid 5%FBS on ice

Conc. (mg/L)	Infectivity (TCID50/mL)
0	6.E+06
0	4.E+07
5	4.E+06
5	2.E+07
10	2.E+06
10	2.E+06
20	4.E+04
20	6.E+04
30	6.E+01
30	4.E+02
50	6.E+01
50	6.E+01

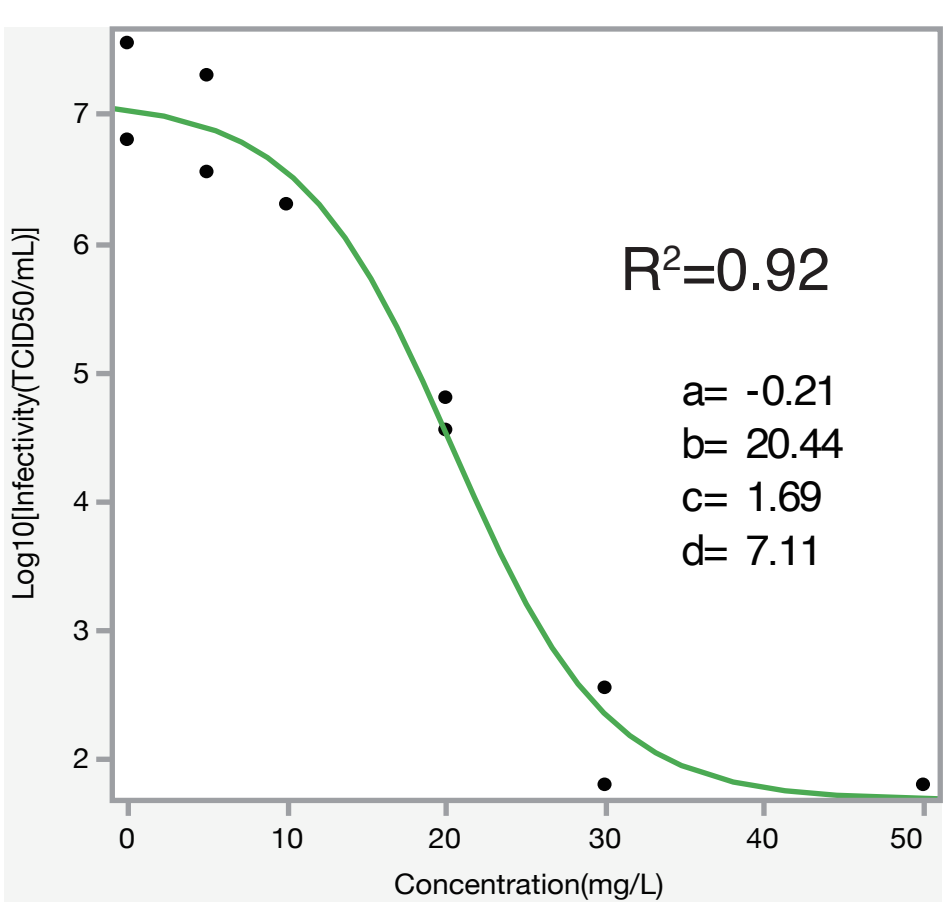

F Sodium hypochlorite 5%FBS on ice

Conc. (mg/L)	Infectivity (TCID50/mL)
0	1.E+07
0	4.E+07
10	2.E+07
20	2.E+04
20	2.E+06
40	4.E+04
50	1.E+03
60	6.E+03
70	1.E+03
80	2.E+03
100	6.E+01
100	1.E+03
150	6.E+01
150	6.E+01
200	6.E+01

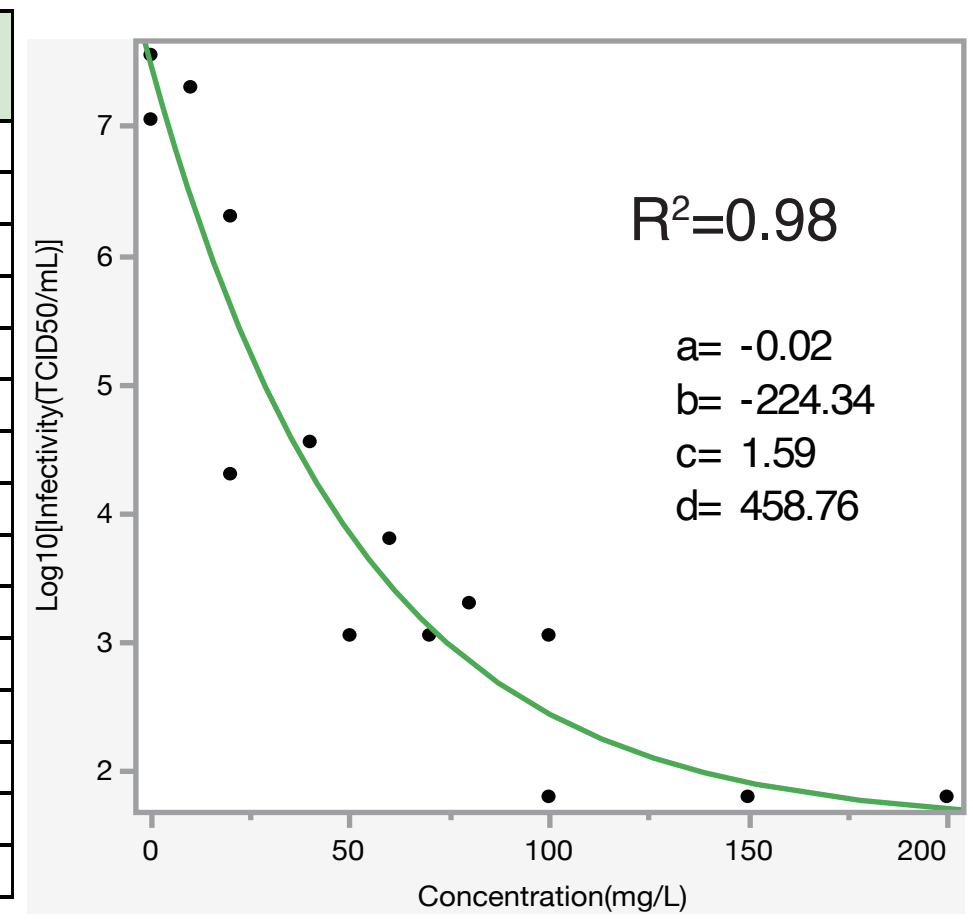

S1 Fig. Concentration–response data and four-parameter logistic regression curves for the suspension tests.

A four-parameter logstic (4PL) model was used. Where y represents the log10 infectivity ($TCID_{50}$/mL) and x denoted the concentration (mg/L). The estimated parameters include growth rate (a), inflection point (b), lower asymptote (c), and upper asymptote (d). The coefficient of determination was expressed as R2. Black circles represent individual observed data points. Analysis was performed using JMP Student Edition.

$$y = c + \frac{d - c}{1 + \exp(-a(x - b))}$$
