# Supplementary material for: Impact of low temperature on the efficacy of chlorous acid water and sodium hypochlorite solution for inactivating avian influenza virus on material surfaces
Source: PLoS One. 2026 Oct 26;21(10):e0360392. doi: 10.1371/journal.pone.0360392 (PMC13704805; doi:10.1371/journal.pone.0360392)
Supplement: Supplementary file 2 [file pone.0360392.s002.pdf]

A Chlorous acid stainless RT

Conc (mg/L)	Infectivity (TCID50/ml)
0	2.E+08
0	1.E+08
0	2.E+07
0	2.E+08
25	6.E+07
50	6.E+05
100	6.E+02
100	6.E+05
100	6.E+04
100	4.E+05
100	4.E+04
100	1.E+04
150	4.E+03
200	6.E+02
200	6.E+02
200	6.E+02

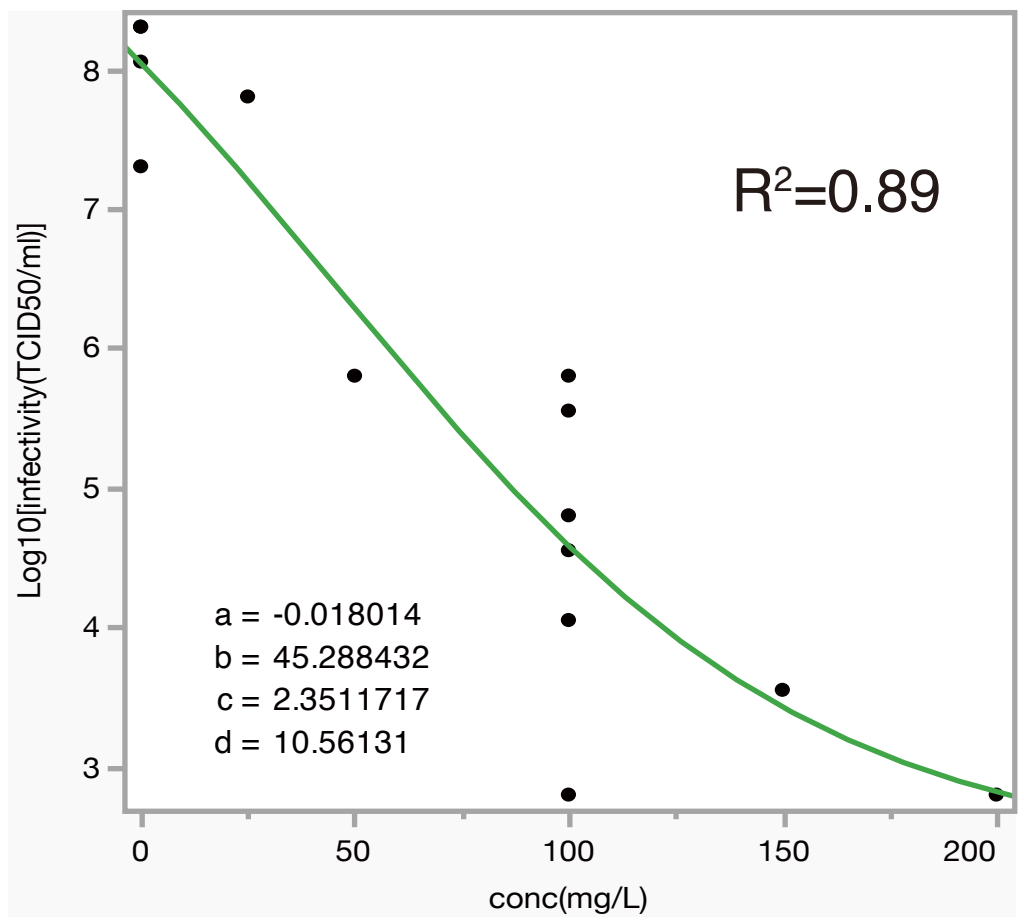

B Sodium hypochlorite stainless RT

Conc (mg/L)	Infectivity (TCID50/ml)
0	1.E+08
0	4.E+07
0	4.E+08
0	6.E+07
100	1.E+06
150	6.E+05
150	2.E+05
200	6.E+01
200	2.E+04
200	6.E+04
250	6.E+01
500	6.E+01

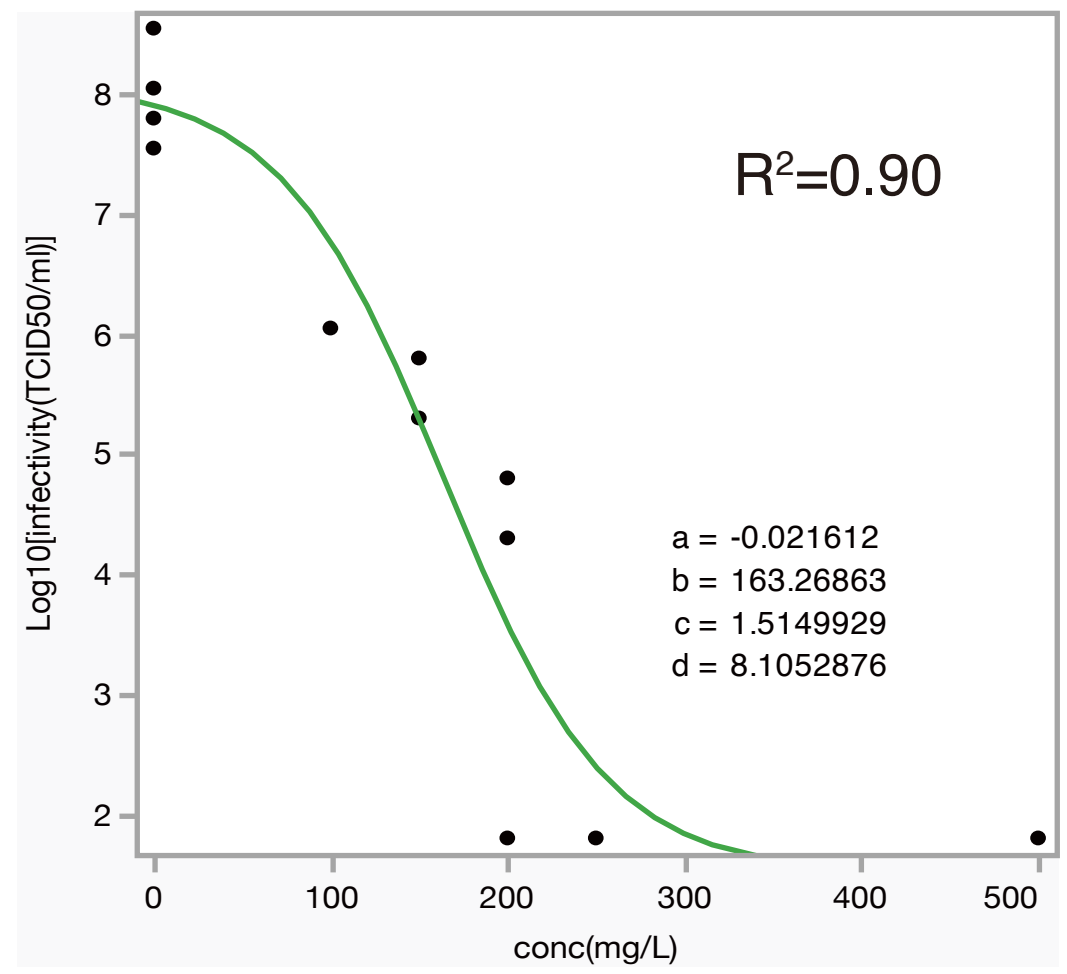

C Chlorous acid plastic RT

Conc (mg/L)	Infectivity (TCID50/ml)
0	4.E+08
0	2.E+08
0	1.E+08
0	4.E+07
0	4.E+07
0	2.E+08
25	1.E+08
50	1.E+07
100	1.E+06
100	6.E+04
100	6.E+03
150	6.E+02
150	1.E+05
150	6.E+02
150	2.E+04
200	6.E+02
200	6.E+02
200	6.E+02
250	6.E+02
250	6.E+02

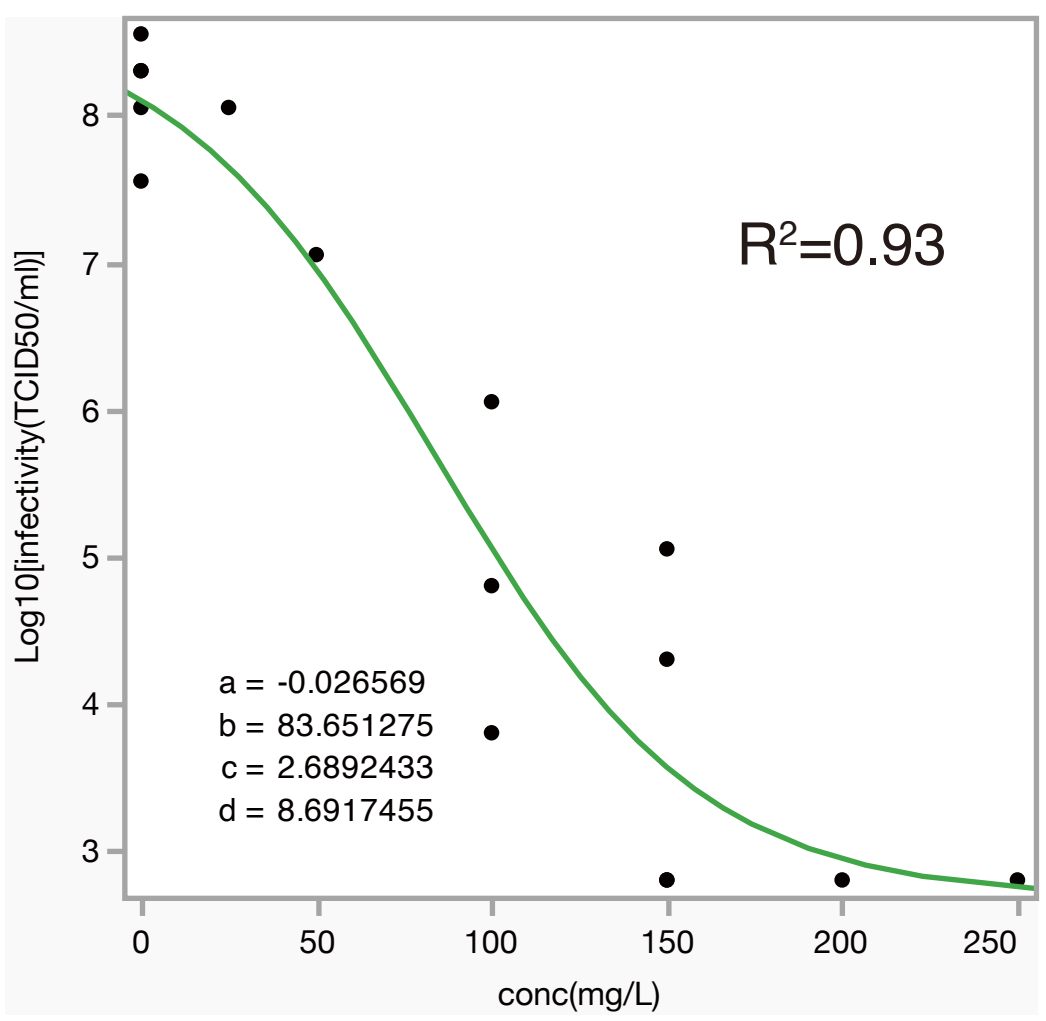

D Sodium hypochlorite plastic RT

Conc (mg/L)	Infectivity (TCID50/ml)
0	6.E+07
0	1.E+08
0	2.E+08
0	1.E+08
100	4.E+06
150	4.E+05
150	6.E+05
200	2.E+04
200	2.E+05
200	6.E+04
250	6.E+01
250	6.E+01
250	4.E+04
250	6.E+01

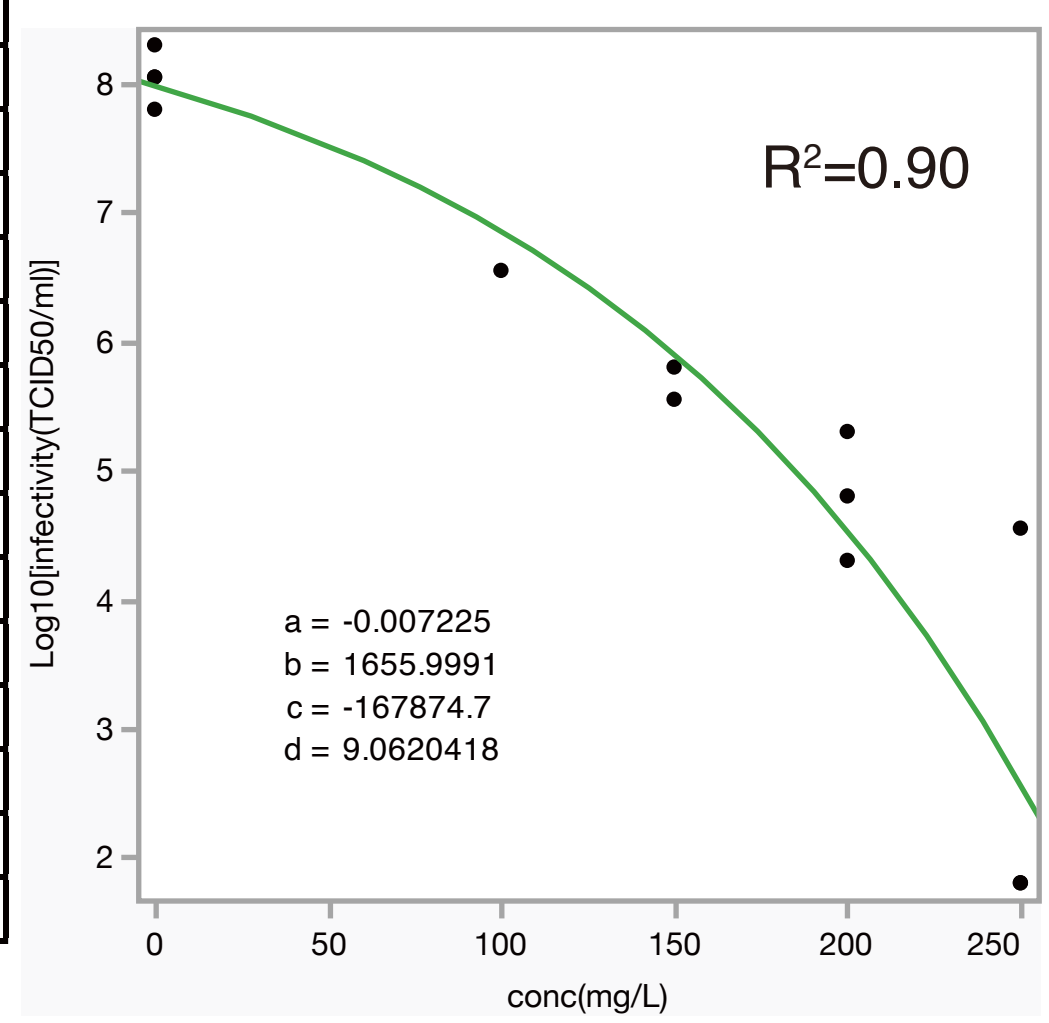

E Chlorous acid rubber RT

Conc (mg/L)	Infectivity (TCID50/ml)
0	1.E+08
0	6.E+07
0	4.E+07
0	1.E+07
100	6.E+04
100	4.E+06
150	1.E+06
150	6.E+03
150	6.E+02
200	6.E+02
200	6.E+02
200	6.E+02

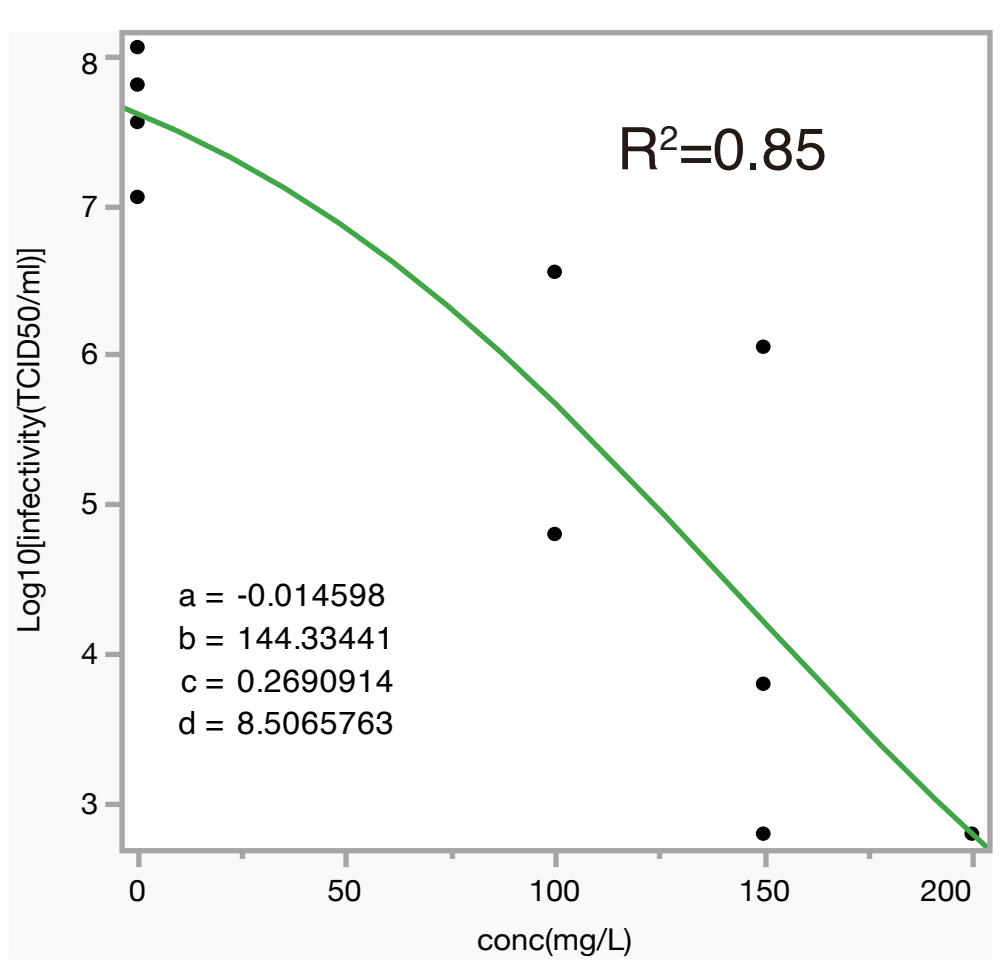

F Sodium hypochlorite rubber RT

Conc (mg/L)	Infectivity (TCID50/ml)
0	4.E+07
0	2.E+07
0	2.E+08
0	4.E+07
0	4.E+07
100	1.E+06
200	1.E+05
200	4.E+05
250	6.E+01
250	1.E+06
250	1.E+06
250	6.E+03
300	6.E+01
300	1.E+06
300	4.E+03
400	4.E+02
500	6.E+01

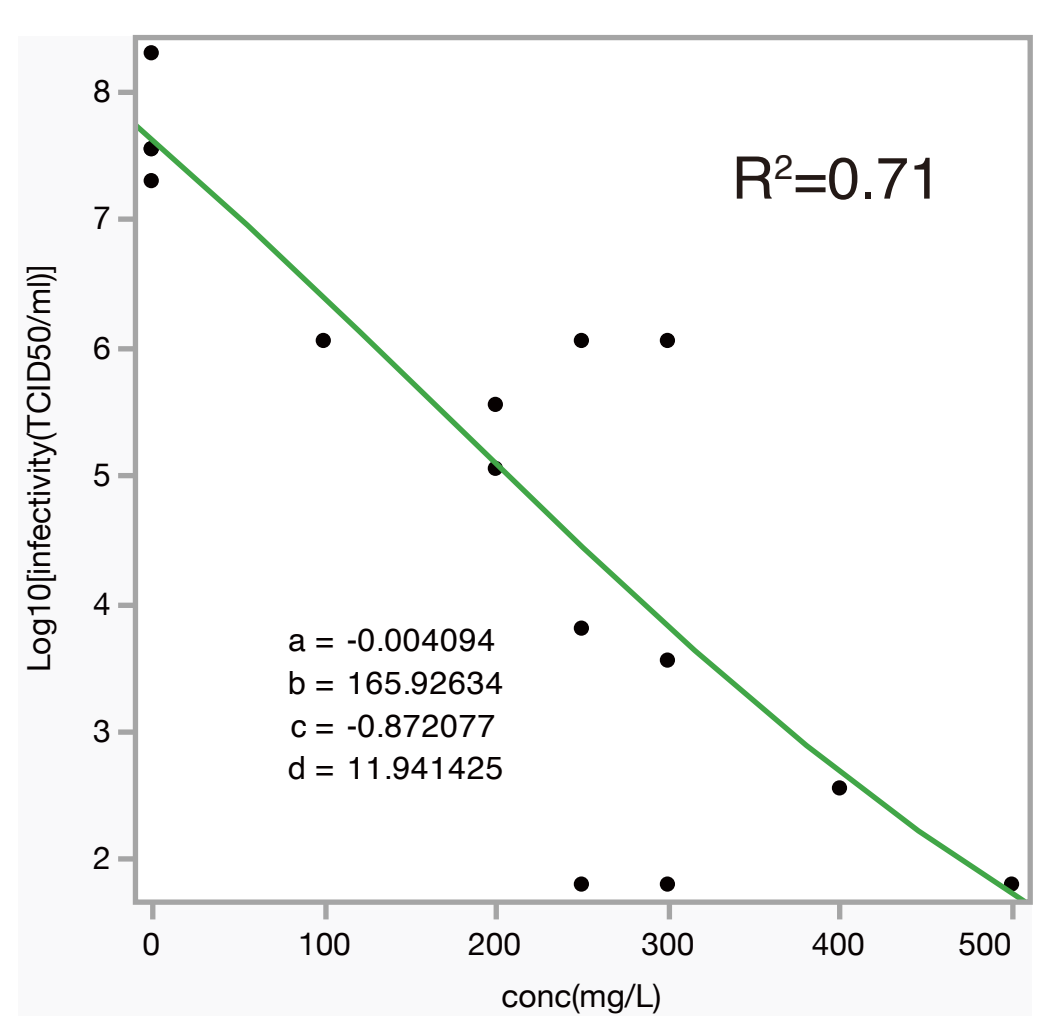

G Chlorous acid stainless on ice

Conc (mg/L)	Infectivity (TCID50/ml)
0	2.E+07
0	6.E+07
0	1.E+08
0	6.E+07
50	6.E+05
50	4.E+04
75	6.E+02
100	6.E+02
125	6.E+02
150	6.E+02
150	6.E+02
150	6.E+02
200	6.E+02

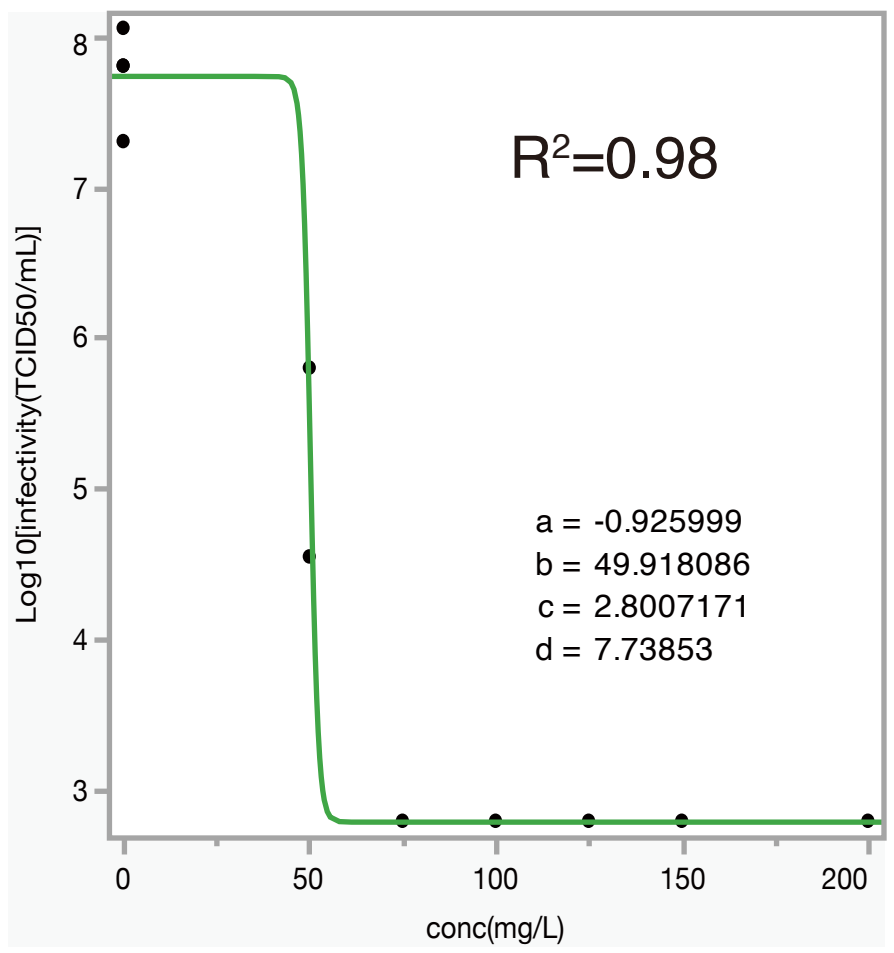

H Sodium hypochlorite stainless on ice

Conc (mg/L)	Infectivity (TCID50/ml)
0	6.E+07
0	1.E+08
0	2.E+08
150	2.E+07
200	2.E+07
250	6.E+06
300	4.E+06
300	1.E+07
400	6.E+04
500	2.E+04
500	6.E+04
500	6.E+03

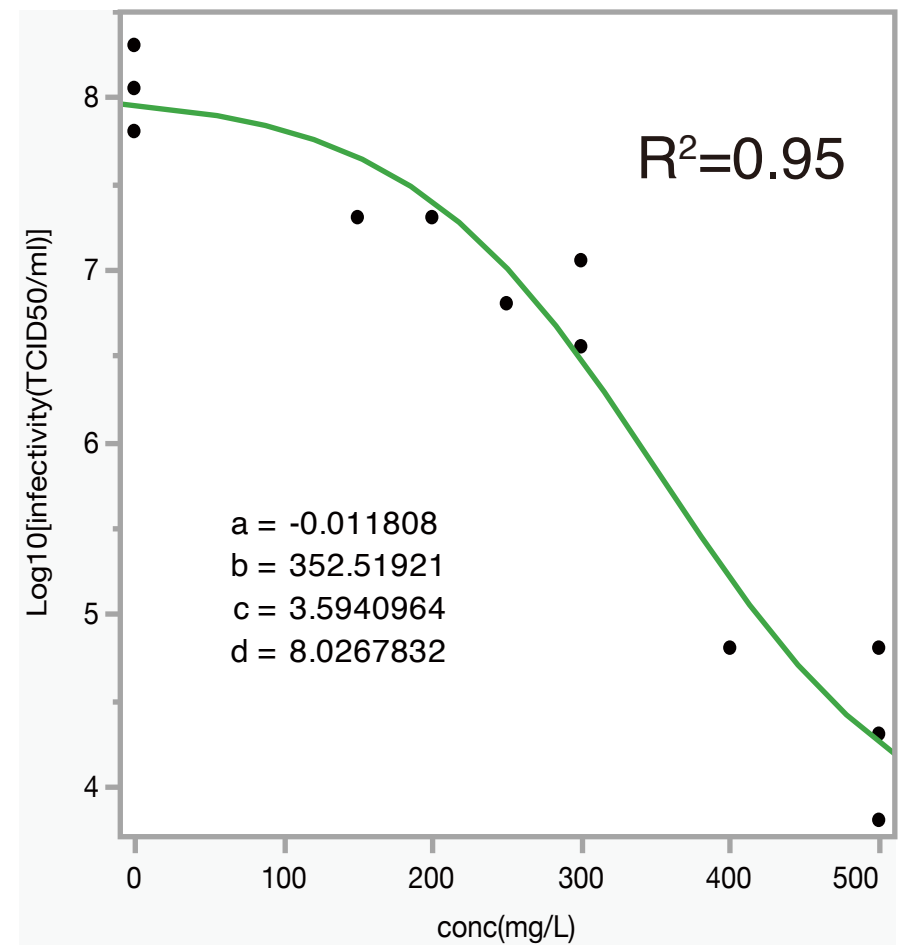

I Chlorous acid plastic on ice

Conc (mg/L)	Infectivity (TCID50/ml)
0	2.E+08
0	2.E+07
0	1.E+07
25	6.E+05
50	6.E+04
50	2.E+04
75	6.E+02
100	6.E+02
100	6.E+02
100	6.E+02
150	6.E+02

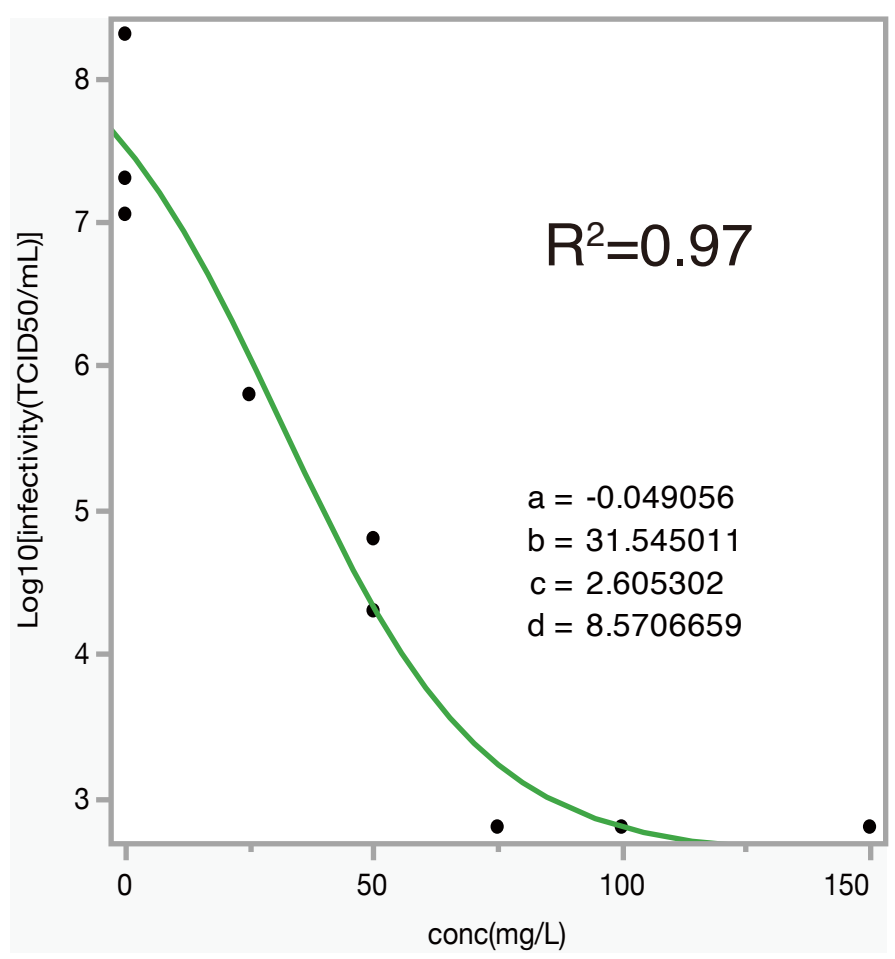

J Sodium hypochlorite plastic on ice

Conc (mg/L)	Infectivity (TCID50/ml)
0	1.E+08
0	4.E+07
0	1.E+08
200	4.E+07
300	1.E+07
400	1.E+06
450	1.E+05
500	1.E+04
500	1.E+04
500	4.E+04

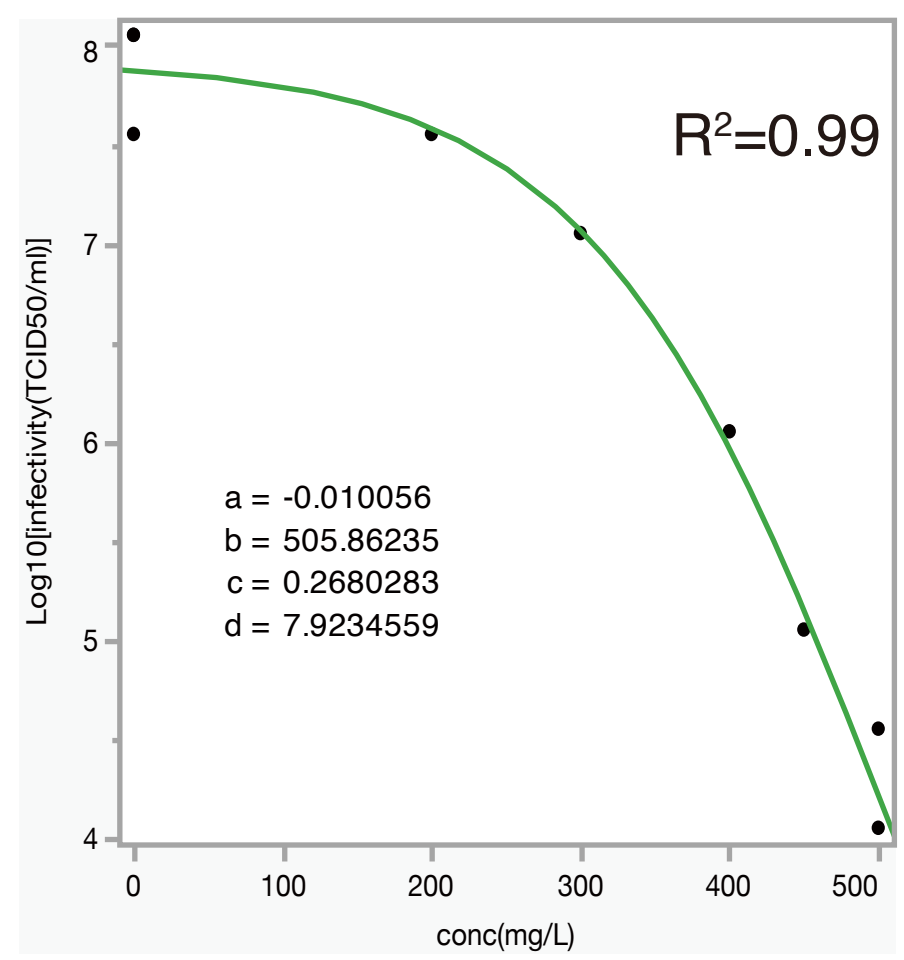

K Chlorous acid rubber on ice

Conc (mg/L)	Infectivity (TCID50/ml)
0	2.E+08
0	1.E+08
0	6.E+07
0	6.E+06
25	1.E+06
50	4.E+05
50	2.E+03
75	6.E+02
100	6.E+02
100	6.E+02
100	6.E+02
150	6.E+02
150	6.E+02
150	6.E+02
200	6.E+02

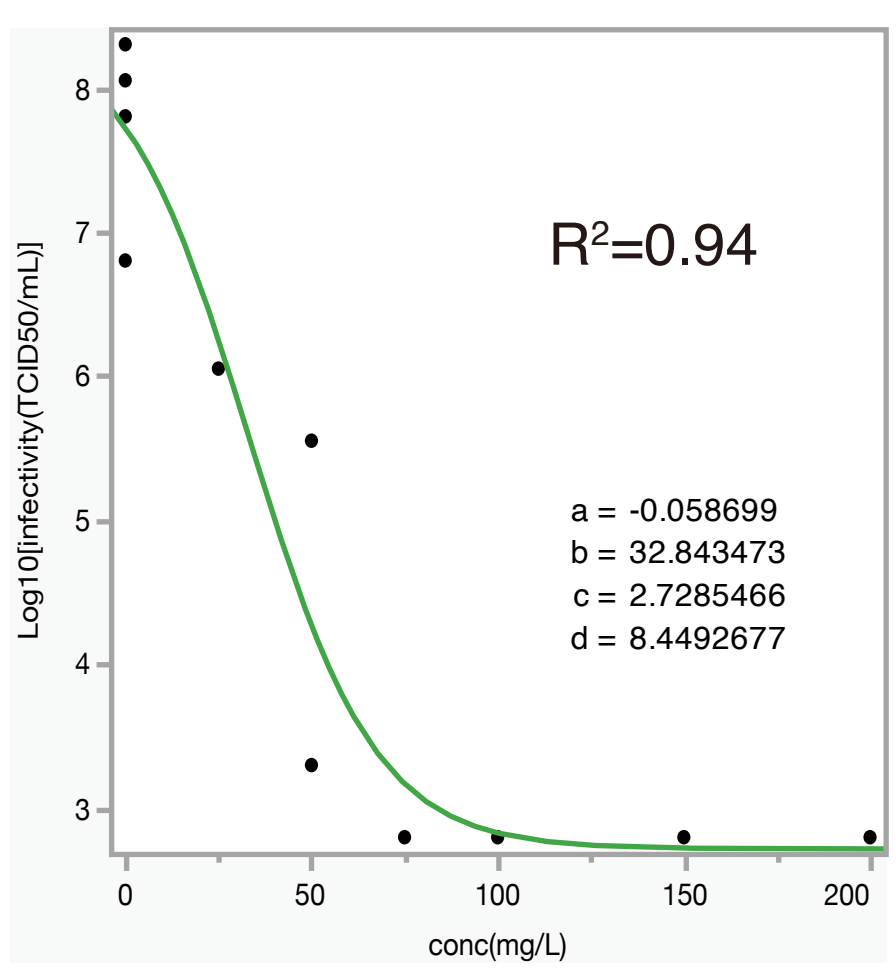

L Sodium hypochlorite rubber on ice

Conc (mg/L)	Infectivity (TCID50/ml)
0	1.E+08
0	2.E+07
0	6.E+07
200	1.E+07
300	6.E+06
400	4.E+06
450	4.E+05
500	1.E+05
500	4.E+04
500	2.E+04

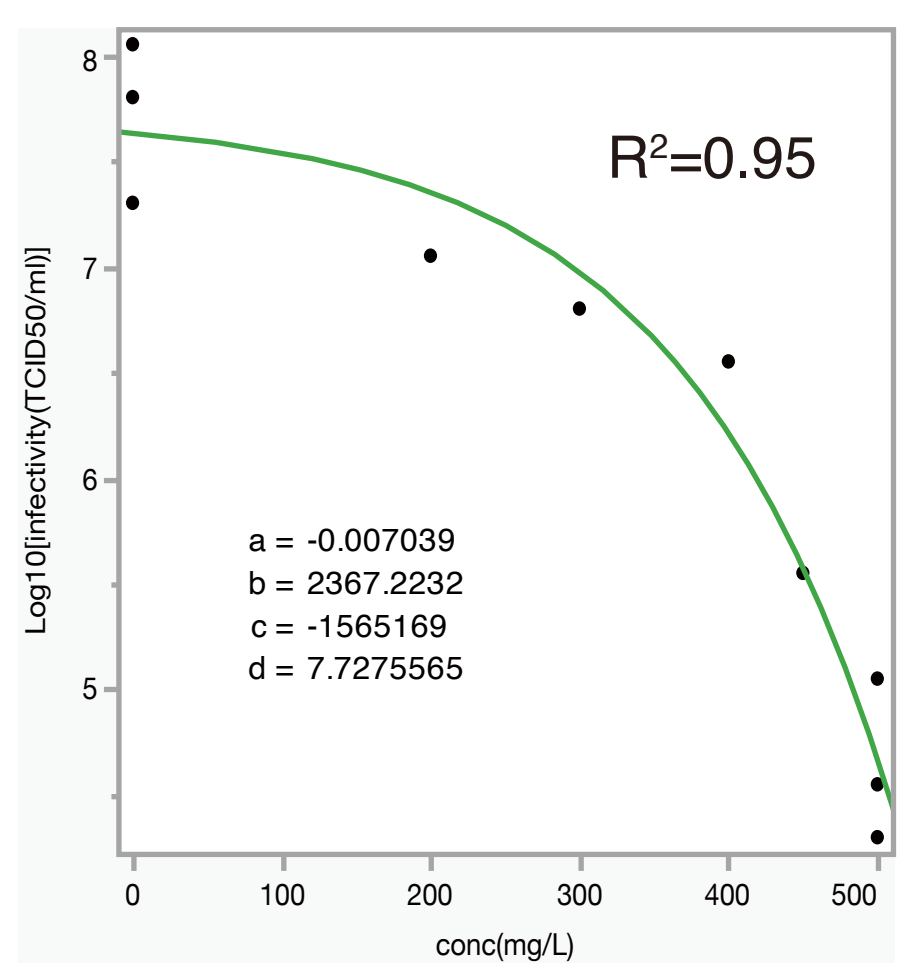

S2 Fig. Concentration–response data and four-parameter logistic regression curves for the surface tests.

A four-parameter logstic (4PL) model was used. Where y represents the log10 infectivity (TCID50/mL) and x denoted the concentration (mg/L). The estimated parameters include growth rate (a), inflection point (b), lower asymptote (c), and upper asymptote (d). The coefficient of determination was expressed as R2. Black circles represent individual observed data points. Analysis was performed using JMP Student Edition.

$$y = c + \frac{d - c}{1 + \exp(-a(x - b))}$$
